# Combined substrate, enzyme and yeast feed in simultaneous saccharification and fermentation allow bioethanol production from pretreated spruce biomass at high solids loadings

**DOI:** 10.1186/1754-6834-7-54

**Published:** 2014-04-08

**Authors:** Rakesh Koppram, Lisbeth Olsson

**Affiliations:** 1 Industrial Biotechnology, Department of Chemical and Biological Engineering, Chalmers University of Technology, Göteborg SE-412 96, Sweden

**Keywords:** High solids, High gravity, *Saccharomyces cerevisiae*, SSF

## Abstract

**Background:**

Economically feasible cellulosic ethanol production requires that the process can be operated at high solid loadings, which currently imparts technical challenges including inefficient mixing leading to heat and mass transfer limitations and high concentrations of inhibitory compounds hindering microbial activity during simultaneous saccharification and fermentation (SSF) process. Consequently, there is a need to develop cost effective processes overcoming the challenges when working at high solid loadings.

**Results:**

In this study we have modified the yeast cultivation procedure and designed a SSF process to address some of the challenges at high water insoluble solids (WIS) content. The slurry of non-detoxified pretreated spruce when used in a batch SSF at 19% (w/w) WIS was found to be inhibitory to *Saccharomyces cerevisiae* Thermosacc that produced 2 g l^-1^ of ethanol. In order to reduce the inhibitory effect, the non-washed solid fraction containing reduced amount of inhibitors compared to the slurry was used in the SSF. Further, the cells were cultivated in the liquid fraction of pretreated spruce in a continuous culture wherein the outflow of cell suspension was used as cell feed to the SSF reactor in order to maintain the metabolic state of the cell. Enhanced cell viability was observed with cell, enzyme and substrate feed in a SSF producing 40 g l^-1^ ethanol after 96 h corresponding to 53% of theoretical yield based on available hexose sugars compared to 28 g l^-1^ ethanol in SSF with enzyme and substrate feed but no cell feed resulting in 37% of theoretical yield at a high solids loading of 20% (w/w) WIS content. The fed-batch SSF also significantly eased the mixing, which is usually challenging in batch SSF at high solids loading.

**Conclusions:**

A simple modification of the cell cultivation procedure together with a combination of yeast, enzyme and substrate feed in a fed-batch SSF process, made it possible to operate at high solids loadings in a conventional bioreactor. The proposed process strategy significantly increased the yeast cell viability and overall ethanol yield. It was also possible to obtain 4% (w/v) ethanol concentration, which is a minimum requirement for an economical distillation process.

## Introduction

Bioethanol produced from lignocellulosic raw materials is considered as a potential transportation fuel providing long-term energy security as well as environmental and economical benefits [1]. Biological conversion of carbohydrates in lignocelluloses to ethanol can be realized by separate hydrolysis and fermentation (SHF) or simultaneous saccharification and fermentation (SSF) of the pretreated raw material. Reduced number of process reactors is one of the features of SSF, which integrates enzymatic hydrolysis and fermentation in one reactor. In SSF, the released sugars from enzymatic hydrolysis are simultaneously consumed by the fermenting microorganism, for example, *Saccharomyces cerevisiae* during fermentation avoiding product inhibition of enzymes and also decreasing the probability of contamination [2]. Distillation of ethanol from the fermentation broth is one of the energy intensive steps [3] and it is crucial to achieve the highest possible ethanol concentration in the fermentation broth, because the cost of distillation decreases with increase in ethanol concentration [4]. Ethanol concentration of 4% (w/v) is the minimal requirement for an economical distillation process. By increasing the water insoluble solids (WIS) concentration in an SSF process, it is possible to achieve high sugar concentration and consequently high final ethanol concentration. Currently, when operating at a high WIS content in conventional stirred tank reactors technological challenges remain, which include high viscosity preventing efficient mixing, high power consumption [5] and high concentrations of lignocelluloses-derived inhibitors [6,7] that inhibit cellulolytic enzymes and metabolism of *S. cerevisiae*. A detailed review of the challenges encountered at high solids loading, its pervasive effect on the pretreatment, enzymatic hydrolysis and fermentation has been presented by Koppram *et al.*[8].

Inhomogeneity caused by inadequate mixing has been previously addressed in several ways. Different reactor designs such as a liquefaction reactor [9] and a simple rotary fermenter [10] have been designed and fabricated and the SSF functionality of these has been demonstrated using pretreated wheat straw with a dry matter content of 32% (w/w) and higher. However, design of specialized reactors is often expensive and may restrict the functionality to a particular feedstock. Alternatively, using conventional stirred-tank reactors several groups have performed SSF in fed-batch mode with substrate and/or enzyme feed [11,12] to overcome challenges when working with high WIS content. A comprehensive review of SSF has been presented by Olofsson *et al*. [13]. Fed-batch SSF has been shown on many occasions to be beneficial for various aspects including: (a) ease of mixing after partial saccharification, resulting in the capacity for more substrate to be added in a stepwise procedure [14]; (b) lower energy consumption due to lower viscosity [5] at any given time point compared to batch SSF; (c) low concentration of inhibitory compounds facilitating the yeast, *S. cerevisiae* to convert them to less inhibitory compounds [15], and (d) maintaining low glucose concentration in the medium, facilitating effective co-consumption of glucose and xylose by recombinant *S. cerevisiae*[16,17]. The potential of fed-batch SSF with substrate and enzyme feed using recombinant xylose utilizing *S. cerevisiae*, at a demo scale of a 10-m^3^ conventional bioreactor, has also been demonstrated using 10% (w/w) WIS content of pretreated corn cobs, producing 4% (w/v) ethanol [18]. Although substrate and enzyme feeding strategies in SSF have been widely explored, the significance of yeast feed in an SSF process remains to be investigated. One of the elemental parts of SSF is the yeast, *S. cerevisiae*, which at high WIS content is subjected to high concentration of inhibitors that affect cell viability [19,20], growth, ethanol yield and productivity [21-23]. Although several detoxification methods can be employed to partly remove the inhibitors [24], the cost of such methods limits their use [25]. Attempts at process modifications to curb the effects of inhibitors have been fruitful. It has been shown that prior to SSF, cultivating the cells in fed-batch mode using the liquid fraction derived from pretreatment improved tolerance towards inhibitors and ethanol productivity in an SSF process using pretreated spruce of relatively low WIS content of 8% (w/w) [26]. However, when working at WIS content as high as 20% (w/w), the severity of inhibition increases and therefore, maintaining the viability of yeast throughout the SSF process becomes crucial. In the present work, we developed a continuous mode of cultivation for adaptation, wherein the outflow of cell suspension from the adaptation reactor was fed to the SSF reactor with the objective of maintaining the robustness of yeast cells during the SSF process. With this mode of yeast feed together with the substrate and enzyme-cocktail feed we evaluated the performance of SSF at 20% (w/w) WIS content using pretreated spruce as biomass. In a parallel study, a mathematical model for the SSF process has been developed and the effect of substrate, enzyme and cell feeding was analyzed (Wang R, Koppram R, Olsson L and Franzén C-J. Modeling and experimental studies of multi-feed simultaneous saccharification and co-fermentation of pretreated birch to ethanol. Manuscript).

## Results and discussion

The aim of the current study was to design an SSF process of high WIS content in a conventional stirred-tank bioreactor. We approached this challenge by using a combination of yeast, enzyme and substrate feed as a means to improve the fermentability at high WIS. In addition, we designed a continuous process for cultivation and adaptation of the yeast stream, to allow optimum performance of the *S. cerevisiae* Thermosacc.

### Evaluation of fermentation performance of Thermosacc

**Figure 1:**
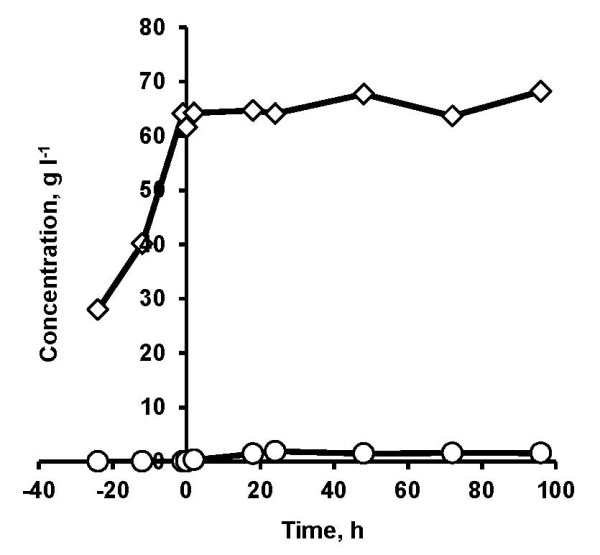
**Evaluation of fermentation performance of Thermosacc in a batch simultaneous saccharification and fermentation (SSF) process using spruce slurry.** Increase in glucose concentration (diamonds) was observed during the initial prehydrolysis for 24 h at 50°C. After 24 h, the temperature was reduced to 35°C and followed by the addition of yeast cell suspension, minute traces of ethanol production (circles) were observed. The enzyme and yeast loading was 7.5 FPU gWIS^-1^ and 5 g L^-1^, respectively. FPU, filter paper units; WIS, water insoluble solids.

The liquid fraction of the pretreated spruce in combination with minimal medium was used in a fed-batch mode for cultivation and adaptation of Thermosacc prior to SSF and anaerobic fermentation. The slurry of pretreated spruce with 20% (w/w) WIS content used in the batch SSF severely affected the fermentability of Thermosacc. The slurry was initially subjected to prehydrolysis for 24 h at 50°C. Prehydrolysis has been shown to reduce the viscosity of the material and also improve overall ethanol yield [27]. During the prehydrolysis period of 24 h, the slurry was sufficiently liquefied to ease mixing, which can be observed from the release of glucose (Figure 1). After 24 h, the temperature was adjusted to 35°C and yeast cell suspension was added to initiate the batch SSF. However, no sugar consumption and ethanol production were observed even after 96 h of SSF (Figure 1).

**Figure 2:**
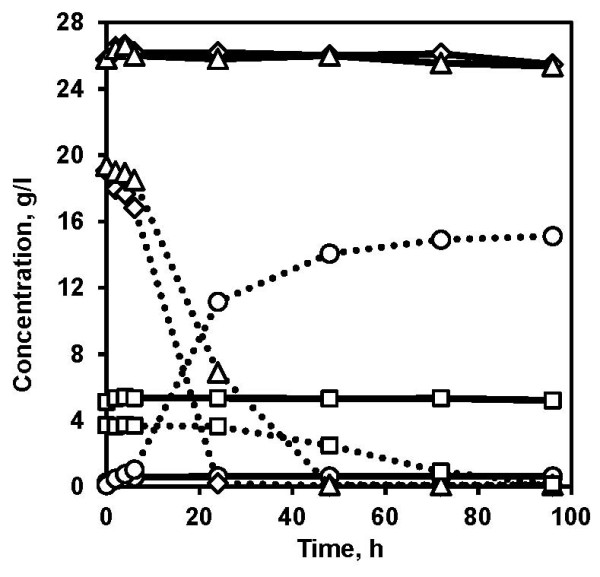
**Evaluation of fermentation performance of Thermosacc in the liquid fraction of pretreated slurry.** Glucose (diamonds), mannose (triangles), and galactose (squares) consumption, and ethanol (circles) production in anaerobic fermentation of liquid fraction diluted to 90% (v/v) (solid lines) and 60% (v/v) (dotted lines) with 3 g L^-1^ of yeast loading. The graph represents the mean of two biological replicates. Error bars are omitted for the sake of clarity.

The liquid fraction with different dilutions was also used in anaerobic fermentations to determine the optimal dilution that promoted fermentation. In the presence of 90% (v/v) of liquid fraction in anaerobic fermentation no hexose sugar consumption and ethanol production were observed even after 96 h (Figure 2). Incidentally, when the liquid fraction was diluted to 60% (v/v), all the glucose and mannose were consumed within 48 h followed by galactose in 96 h, reaching a final ethanol concentration of 15 g L^-1^ (Figure 2) corresponding to 70% of the theoretical ethanol yield. Although, fed-batch adaptation of yeast using the liquid fraction did not contribute to fermentation in batch SSF of spruce slurry, reducing the inhibitors by diluting the liquid fraction in anaerobic fermentation significantly improved the fermentation performance. However, dilution contributes to increased water consumption and may not be a viable option from an industrial perspective. As most of the inhibitors are water-soluble, a significant fraction of inhibitors can be removed by separating the solid and liquid fraction. The non-washed solid fraction obtained by centrifugation of pretreated slurry contained reduced concentration of inhibitors compared to the slurry (Table 1). Also, the concentrations of inhibitors in the non-washed solid fraction were similar to the concentrations in the 60% (v/v) liquid fraction used in the anaerobic fermentation that showed improved fermentation performance. Although the separation of pretreated slurry into liquid and solid fraction is also an energy-consuming process, it could nevertheless be essential, mainly for three reasons: (1) to use the liquid fraction to cultivate and adapt the yeast prior to SSF; (2) to reduce the inhibitor concentration in the solid fraction, which can then be used as substrate for SSF, and (3) to increase the WIS content in the solid fraction, which gives flexibility to operate the process with more than 20% (w/w) WIS content, which is not possible with the whole slurry obtained with the pretreatment method used in the study unless evaporation is employed.

**Table 1:** Composition of spruce slurry and non-washed solids used in simultaneous saccharification and fermentation (SSF)

**Components**	**Concentration when slurry was used, g kg**^-1*^	**Concentration when non-washed solids were used, g kg**^-1*^
WIS	200	200
Glucan	106	106
Glucose	24.8	14.5
Mannose	24.6	14.4
Galactose	4.9	2.9
Xylose	11.9	6.9
HMF	2.8	1.7
Furfural	2.3	1.3
Acetic acid	5.7	3.3

*Concentrations correspond to a total water insoluble solids (WIS) content of 20% (w/w) in SSF with a total working weight of 1.5 kg. HMF, 5-hydroxy-methyl furfural.

### SSF with the solid fraction

#### High WIS content in batch SSF causes inhomogeneity

**Figure 3:**
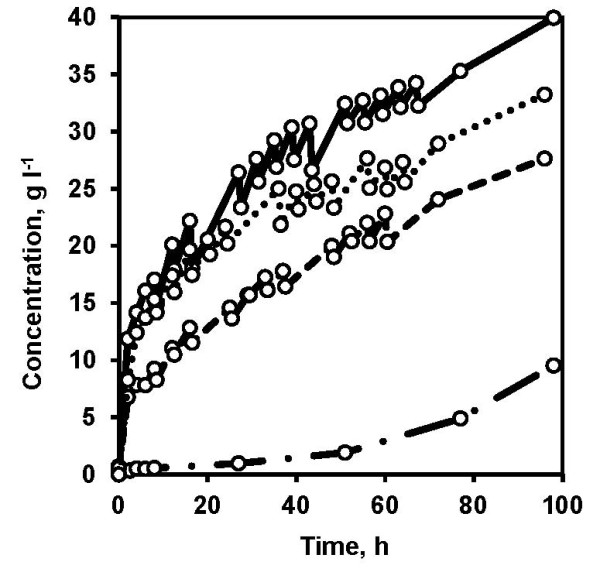
**Comparison of ethanol production during batch and fed-batch simultaneous saccharification and fermentation (SSF) using non-washed solids as substrate.** Ethanol concentration (circles) during batch SSF (dashed-dotted lines), fed-batch SSF with substrate and enzyme feed (dashed lines), fed-batch SSF with yeast and substrate feed (dotted lines) and fed-batch SSF with yeast, enzymes and substrate feed (solid lines). The graph represents the mean of two biological replicates. Error bars are omitted for the sake of clarity. The total water insoluble solids (WIS), enzyme loading and yeast loading were 20% (w/w), 7.5 filter paper units (FPU) g WIS^-1^ and 5 g L^-1^, respectively, in all the experiments.

The solid fraction obtained after centrifugation of pretreated slurry contained increased WIS content of 30% (w/w) and reduced concentration of inhibitors (Table 1) compared to the pretreated slurry with a WIS content of 20% (w/w). The fermentability of the solid fraction was assessed in batch SSF at 20% (w/w) WIS content. One of the major problems encountered during the batch SSF was difficulty in mixing, and as a result, local variations in temperature (35 ± 4°C) were encountered. Sampling was unconventionally done during the batch SSF by opening the fermenter lid and manually mixing the contents with a spatula, and a small portion was then withdrawn. There was a 40-h delayed onset of glucose consumption and after 96 h, only free glucose and mannose present in the solid fraction were consumed and the ethanol concentration reached 9.5 g L^-1^ (Figure 3) corresponding to 13% of the theoretical ethanol yield based on available hexose sugars. The solid fraction remained unhydrolyzed at the end of 96 h and no glucose was detected in the medium at this time point, which possibly indicates that the enzymes were no longer active. This can be explained by delayed stabilization of pH caused by difficulty in mixing, and therefore the high local pH possibly caused the inactivation of enzymes. Although this clearly indicates the existence of inhomogeneity in batch SSF at high WIS content, in the next step we evaluated the possibility to use different fed-batch strategies to improve the process outcome at high WIS content.

#### Yeast feed improves the overall ethanol yield in SSF at high WIS content

**Figure 4:**
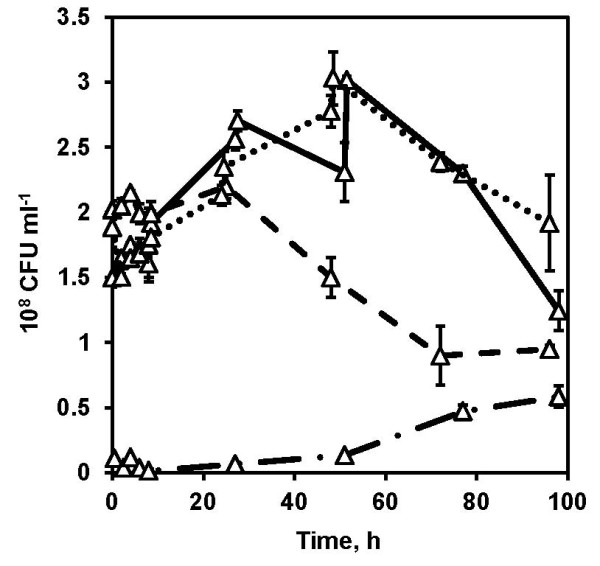
**Comparison of colony forming units (CFU) during the batch and fed-batch SSF using non-washed solids as substrate.** CFU (triangles) during batch SSF (dashed-dotted lines), fed-batch SSF with substrate and enzyme feed (dashed lines), fed-batch SSF with yeast and substrate feed (dotted lines) and fed-batch SSF with yeast, enzymes and substrate feed (solid lines). The graph represents the mean of two biological replicates and two technical replicates with error bars indicating standard deviation. The total WIS, enzyme loading and yeast loading were 20% (w/w), 7.5 FPU gWIS^-1^ and 5 g L^-1^, respectively, in all the experiments.

The interplay between substrate, enzyme and yeast forms a basis for optimizing the SSF process. Several combinations to feed the substrate, enzyme preparation and yeast are possible in fed-batch SSF. Primarily, it is important to feed the substrate when working at high WIS content to improve the ethanol yield, as shown by previous studies [28,29]. Therefore, substrate feed remained as a minimum requirement in the current study. Together with the substrate feed the combination of enzyme and/or cell feed was also investigated (Table 2). Irrespective of the combination of feeding, the fed-batch SSF significantly improved the mixing, even though the stirrer speed was set at 400 rpm compared to 700 rpm in the batch SSF. This could be directly translated to lower energy-consumption for mixing, which is one of the crucial factors for an economical process. The total WIS, enzyme and yeast load in fed-batch SSF were the same as in the batch SSF. However, we initially operated the fed-batch SSF at relatively low WIS content and high enzyme and yeast loading per gram of WIS content. This significantly contributed to a well-mixed process. Sampling was no longer an inconvenient step because the medium was able to be drawn through the regular sampling port, which significantly decreased the contamination risk. The yeast cultivation procedure significantly affected the fermentation performance. When the cells were cultivated in fed-batch mode using the liquid fraction and added at once to the fed-batch SSF with substrate and enzyme feed, an overall ethanol yield of 37% of the theoretical maximum based on available hexose sugars was achieved. However, when the cells were cultivated in a continuous mode using the liquid fraction and fed to the SSF process with substrate feed, the overall ethanol yield was significantly increased to 44% and 53% of the theoretical maximum based on available hexose sugars with no enzyme feed and in combination with enzyme feed, respectively. The increased ethanol yield can be clearly attributed to the increased yeast viability (Figure 4) measured by counting the colonies on yeast extract peptone dextrose (YPD) plates. Higher ethanol productivity (Figure 3) and increased cell viability (Figure 4) were observed in a fed-batch SSF experiment involving feeding with continuous-mode adapted yeast compared to the fed-batch SSF experiment with fed-batch adapted yeast added all at once at the start of SSF. It can be speculated that in order to maintain the cell viability it is important to maintain the cells in a robust metabolic state, a feature that can be achieved by continuous culture as it ensures steady physicochemical conditions in the bioreactor and therefore, a physiological steady-state can theoretically be achieved [30]. It is also known that a constant product (cells) quality can be maintained in a continuous culture for a relatively longer time compared to the batch culture. Furthermore during the continuous cultivation, the concentration of inhibitors such as acetic acid, 5-hydroxy-methyl furfural (HMF) and furfural were found to be near zero. This indicates that the biological conversion and detoxification of inhibitors are key aspects during the adaptation step. Therefore, adapting the cells in a fed-batch mode also to increase the cell concentration and switching the culture to a continuous mode for cell feeding in a fed-batch SSF can be advantageous from a cell viability point-of-view, especially when working at high solid loadings. Our parallel study on mathematical modeling of the process also strongly indicated increased yeast viability as an important indicator for the overall improved ethanol yield. In addition, the model also indicated increased xylose consumption when xylose-rich feedstock was employed in the multi-feed SSF process (Wang R, Koppram R, Olsson L and Franzén C-J. Modeling and experimental studies of multi-feed simultaneous saccharification and co-fermentation of pretreated birch to ethanol. Manuscript). It has been shown that filling a 10 m^3^ reactor with substrate before beginning the batch SSF can be a time-consuming process [18]. However, this multi-feed SSF can be considered feasible since the process commences with the onset of substrate, enzymes and yeast feed, which can collectively reduce the overall process time. Therefore, from an industrial perspective fed-batch SSF could be an attractive feature. However, a techno-economic evaluation is needed to determine the impact of such a strategy on overall process economy, since fed-batch or a continuous mode of operation beckons good process control and associated costs.

**Table 2:** Summary of simultaneous saccharification and fermentation (SSF) experiments

**SSF**	**Substrate**	**Water insoluble solids (WIS)**		**Yeast loading amount, g**	**Enzyme loading, FPU**	**Glucose**^**e**^**, g**	**Ethanol**	
		**% (w/w)**	**Amount, g**				**g L**^**-1**^	**%**^**f**^
B^*^	Slurry	19	285	7.5	2138	68.0	2.1	2
B	Solid fraction^a^	20	300	7.5	2250	0.1	9.5	13
FB	Solid fraction^a^	10 to 20^b^	50 to 300	7.5	1125 to 2250^d^	0.1	27.9	37
FB	Solid fraction^a^	10 to 20^b^	50 to 300	3.5 to 7.5^c^	2250	0.5	33	44
FB	Solid fraction^a^	10 to 20^b^	50 to 300	3.5 to 7.5^c^	1125 to 2250^d^	0.5	40	53

*Batch SSF was carried out with prehydrolysis of 24 h. ^a^The solid fraction with a WIS content of 31% (w/w) was not washed and therefore, free sugars and inhibitors exist in it but in lower amounts compared to the slurry. ^b^Fed-batch experiments started as a batch at 10% WIS with a working weight of 500 g. After 8 h, substrate was fed every 4 or 8 h until 65 h to a final WIS content of 20%. ^c^Fed-batch experiments started as a batch with an initial yeast loading of 3.5 g (7 g L^-1^). After 8 h, cell suspension was fed every 4 or 8 h until 65 h to a final yeast amount of 7.5 g (5 g L^-1^). ^d^Fed-batch experiments started as a batch with an initial enzyme loading of 1125 filter paper units (FPU) (22.5 FPU g of WIS^-1^). After 8 h, enzyme preparation was fed every 4 or 8 h until 65 h to a final enzyme loading of 2250 FPU (7.5 FPU g of WIS^-1^). ^e^Residual amounts of glucose at the end of 96 h of SSF. ^f^Yield of theoretical maximum based on the total amount of glucose, mannose and galactose in the pretreated spruce. Yields are calculated for the concentrations after 96 h of SSF.

Although the substrate feed improved the mixing and cell feed improved the cell viability, the highest observed ethanol yield was only 53% of the theoretical maximum despite the fact that a large fraction of cells remained viable even after 96 h of fed-batch SSF (Figure 4). The concentration levels of hexose sugars including glucose, mannose and galactose remained below 0.3 g L^-1^ after 96 h, indicating that there were no fermentable monomeric sugars. This likely indicates that enzymatic hydrolysis could be a possible limiting factor affecting the overall ethanol yield. It has previously been shown that at increasing solids concentration, the proportion of adsorbed cellulase decreased because of adsorption inhibition, the mechanism of which remains elusive [31]. Also, there is previous evidence that xylose and xylooligomers with a concentration as low as 1.67 g L^-1^ can decrease enzymatic hydrolysis rates and yields [32]. In our study xylose was not fermentable by Thermosacc and the existence of xylose at a significant concentration of 15 g L^-1^ in the SSF can hinder enzymatic hydrolysis. Besides, softwoods, such as spruce, contain relatively high lignin content [13], which can cause increased nonspecific adsorption of cellulases to lignin [32] especially at high WIS content. Evidence also suggests that the simple and oligomeric phenolics generated during pretreatment, cause inhibition and precipitation of enzymes, even at low concentrations [6,33]. Furthermore, the operation of SSF at suboptimal temperature of enzymes combined with other aforementioned factors are some of the areas that need improvement to further increase the ethanol yields to make the process economically feasible.

## Conclusion

We here demonstrated that the cultivation of yeast in a continuous culture wherein the outflow of cell suspension was fed to the SSF reactor, can significantly enhance cell viability and contribute to overall increase in ethanol yield. We also show that it is possible to work at a high WIS content of 20% (w/w) in conventional bioreactors using a well-designed fed-batch SSF process. Furthermore, we demonstrated the production of high ethanol-concentration (40 g L^-1^), which is the minimal requirement for an economical distillation process. In addition, the potential of fed-batch SSF with substrate, enzyme and yeast feed can be improved by addressing the challenges pertaining to enzymatic hydrolysis.

## Methods

### Media

The inoculum for anaerobic fermentations and SSF experiments was prepared by cultivation in minimal medium containing 20 g L^-1^ glucose and enriched with salts, and 2-fold addition of vitamins and trace elements according to Verduyn *et al.*[34]. The pH of the medium was set to 6.0 with 1 M NaOH for all shake-flask cultivations. YPD plates containing 10 g L^-1^ yeast extract, 20 g L^-1^ peptone, 20 g L^-1^ glucose and 20 g L^-1^ agar were used for colony forming unit (CFU) determination during the SSF. A YNB plate containing 6.9 g L^-1^ yeast nitrogen base (without amino acids), 20 g L^-1^ glucose and 20 g L^-1^ agar was used to isolate individual colonies of yeast.

### Microorganism

*S. cerevisiae* Thermosacc Dry was purchased from Lallemand, USA. The dry yeast was suspended in 5 ml of minimal medium and a loop full of cell suspension was streaked on a YNB plate, which was later incubated at 30°C for 2 days. A loop full of colonies of the same size were picked and re-suspended in 50 ml of minimal medium in a 150-ml shake flask, which was later incubated at 30°C on an orbital shaker set at 180 rpm until the late exponential phase (approximately 20 h). Aliquots of cell suspension (1 ml) were mixed with 0.5 ml of 60% sterile glycerol and stored at -80°C in sterile vials. Volumes of 100 μl from the vials were used to inoculate precultures.

### Pretreated spruce

Spruce slurry with a WIS content of 20.3%, w/w (weight of insoluble solids to weight of slurry) was received from SEKAB-E-Technology AB (Örnsköldsvik, Sweden) and the composition of slurry is given in Table 1. The slurry was centrifuged at 10,000 g for 10 minutes to separate the solid and the liquid fractions. Neither the solid nor the liquid fraction was subjected to chemical or physical detoxification. The solid fraction was used as a substrate feed for SSF. The liquid fraction along with the minimal medium was used for cultivation during the adaptation step. The liquid fraction was also used for anaerobic fermentation to assess its fermentability by Thermosacc.

### Cultivation of Thermosacc

The preculture for cell cultivation was developed in 50 ml of minimal medium in a 150-ml shake flask incubated at 30°C on an orbital shaker set at 180 rpm for 20 h. The cells were cultivated in a 3.6-L Infors HT-Labfors bioreactor in two stages, an initial batch phase in minimal medium, followed by a fed-batch phase of adaptation in a medium of liquid fraction with minimal medium. The batch phase was initiated by adding 50 ml of preculture to a working volume of 500 ml, and the cultivation was carried out until the growth on glucose followed by ethanol was completed, which was indicated by CO_2_ evolution in the off-gas and by the dissolved oxygen concentration in the culture. Upon exhaustion of glucose and ethanol in the batch phase, a feed solution of the liquid fraction (from pretreated slurry) and minimal medium was fed linearly for 16 h to a final volume of 1.3 L. The concentration of liquid fraction in the feed solution was 50% (v/v). The minimal medium was supplemented to the feed solution to a final hexose (glucose, mannose and galactose) concentration of 50 g L^-1^ and the salts, vitamins and trace elements were correspondingly scaled up. The stirrer speed was set to 700 rpm during the batch phase and increased linearly to 1,000 rpm during the fed-batch phase. The aeration rate was maintained at 1 volume per volume per minute (vvm) and the pH was maintained at 5.0 by automatic addition of 2 M NaOH. Cell suspension was harvested after the fed-batch phase and used as yeast loading for anaerobic fermentation and also as initial yeast loading for the subsequent fed-batch SSF process. After the fed-batch phase the cultivation process was changed to a continuous mode with the adaptation feed solution with a flow rate of 50 ml h^-1^ and the working volume was maintained at 1 L. A part of the outflow was manually regulated as a cell suspension feed to the SSF reactor.

### Anaerobic fermentation in shake flasks

The fermentation was carried out to a working volume of 50 ml in 100-ml shake flasks fitted with a glycerol loop providing an anaerobic condition. Different concentrations of the liquid fraction (90%, 60% and 40% (v/v)) were screened. The pH of the liquid fraction was adjusted to 6.0 and supplemented with 0.5 g l^-1^ (NH_4_)_2_HPO_4_, 125 ppm of vitahop (Betatech Gmbh, Schwabach, Germany) (to suppress bacterial growth). The fermentation was initiated by adding harvested cell suspension to reach a yeast concentration of 3 g dry weight L^-1^. The flasks were incubated at 30°C on an orbital shaker set at 180 rpm for 96 h and samples were withdrawn for optical density (OD)_650_ measurement and extracellular metabolite analysis.

### SSF

All the SSF experiments were carried out to a total WIS content of 20% (w/w), total enzyme loading of 7.5 FPU g^-1^ WIS, total yeast loading of 5 g dry weight L^-1^ and to a final working weight of 1.5 kg in 3.6-L Infors HT-Labfors reactors. An enzyme preparation, Cellic Ctec-2 from Novozymes A/S, Denmark with a filter paper activity of 149 FPU g^-1^ enzyme, β-glucosidase activity of 590 IU g^-1^ enzyme was used. The solid fraction of the pretreated spruce was used as the substrate, and the pH of this was adjusted to 5.0 using 10 M NaOH and supplemented with 0.5 g L^-1^ (NH_4_)_2_HPO_4_ and 125 ppm of vitahop. Batch SSF was initiated by adding fed-batch adapted cell suspension and enzyme preparation. Fed-batch SSF was initially started as a batch SSF of 500 g as a working weight with 10% (w/w) WIS content, 50% of total yeast cell suspension (fed-batch adapted) and 50% of total enzyme preparation. After an initial period of 8 h of batch SSF the solid fraction, enzyme preparation and yeast cell suspension (continuous mode adapted) were manually fed every 4 or 8 h. The manual feeding was carried out for a period of 65 h. Fed-batch SSF with substrate and enzyme-cocktail feed was carried out in a similar way but with all the required yeast cell suspension added at the beginning. Fed-batch SSF with substrate and cell-suspension feed was carried out in a similar manner but with all the required enzyme preparation added at the beginning. The temperature was maintained at 35°C and pH at 5.0 by automatic addition of 5 M NaOH. The stirrer speed was set at 700 rpm for batch SSF and 400 rpm for fed-batch SSF. All the experiments were carried out in duplicates.

### Colony-forming units

Samples collected during the SSF were serially diluted using sterile normal saline solution; 100 μl of two of the dilutions were plated on YPD plates and incubated at 30°C for 2 days and the colonies were counted and represented as CFU ml^-1^.

### Sample preparation

To make the sample pipettable equivalent to water, 1 g of the withdrawn sample from SSF was diluted five times with milliQ water. The diluted samples were quantified for metabolites by HPLC. The concentrations (including the dilution factor) of metabolites obtained by HPLC were represented in g (metabolite) kg (slurry)^-1^. This concentration was used to determine the yields presented in Table 2. However, for the data representation in the graphs and elsewhere in the text, the concentration (g kg^-1^) was converted to concentration (g L^-1^) by multiplying with a conversion factor of 1.06 (kg L^-1^). The conversion factor was determined by pipetting 1 ml of slurry using a cut tip and weighing.

### Analysis of metabolites

Samples for extracellular metabolites were analyzed by HPLC using Aminex HPX-87H column with 30 × 4.6 mm Cation-H Biorad micro-guard column (Bio-Rad Laboratories AB, Solna, Sweden) maintained at 45°C; 5 mM H_2_SO_4_ was used as an eluent at a flow rate of 0.6 ml min^-1^. Glycerol, ethanol and acetic acid were detected using an RI detector maintained at 35°C; HMF and furfural were detected using a UV detector at 210 nm. The monomeric sugars in the samples were analyzed by high performance anion exchange chromatography using 4 × 250 mm Dionex CarboPac PA1 column with 4 × 50 mm guard column (Thermo Scientific, Sweden) maintained at 30°C. Eluent A: 300 mM NaOH, eluent B: 100 mM NaOH + 85 mM sodium acetate were used for elution at a flow rate of 1 ml min^-1^. Monosaccharides including galactose, glucose and mannose were detected using pulsed amperometric detector.

### Ethanol yield calculation

The ethanol yield was represented as % of the maximum theoretical yield based on total available hexose sugars. The sum of available fermentable sugars, including glucose, mannose and galactose in the liquid fraction and WIS fraction was calculated. Due to the addition of water during hydrolysis, the theoretical weight of glucose released is 1.11 times the weight of glucan. By using the maximum theoretical ethanol yield of 0.51 g g^-1^ sugar, the maximum ethanol that can be produced from total available sugars was calculated. The percentage of the theoretical ethanol yield is defined as:

$$\begin{array}{ll} Y_{\mathrm{SE}}= & 100^{*}\mathrm{produced}\;\mathrm{amount}\;\mathrm{of}\;\mathrm{ethanol}\;\left(g\right)/\\ & \mathrm{maximum}\;\mathrm{theoretical}\;\mathrm{amount}\;\mathrm{of}\;\mathrm{ethanol}\;\left(g\right) \end{array}$$

## Abbreviations

CFU: colony-forming units; FPU: filter paper units; HMF: 5-hydroxy-methyl furfural; HPAEC: high performance anion exchange chromatography; HPLC: high performance liquid chromatography; SSF: simultaneous saccharification and fermentation; WIS: water insoluble solids; YPD: yeast extract peptone dextrose.

## Competing interests

The authors declare no conflicts of interest.

## Authors’ contributions

RK and LO were involved in the conception and design of the study. RK performed the experimental work. RK and LO critically analyzed the data. RK wrote the manuscript. Both authors commented on the manuscript, and read and approved the final manuscript.

## References

[B1] Balat M Production of bioethanol from lignocellulosic materials via the biochemical pathway: a review Energ Convers Manage 2011 52 858 875 10.1016/j.enconman.2010.08.013

[B2] Galbe M Zacchi G A review of the production of ethanol from softwood Appl Microbiol Biotechnol 2002 59 618 628 10.1007/s00253-002-1058-9 12226717

[B3] Wingren A Galbe M Zacchi G Techno-economic evaluation of producing ethanol from softwood: comparison of SSF and SHF and identification of bottlenecks Biotechnol Prog 2003 19 1109 1117 12892470 10.1021/bp0340180

[B4] Zacchi G Axelsson A Economic-evaluation of preconcentration in production of ethanol from dilute sugar solutions Biotechnol Bioeng 1989 34 223 233 10.1002/bit.260340211 18588096

[B5] Dasari RK Dunaway K Berson RE A scraped surface bioreactor for enzymatic saccharification of pretreated corn stover slurries Energ Fuel 2009 23 492 497 10.1021/ef800434u

[B6] Kim Y Ximenes E Mosier NS Ladisch MR Soluble inhibitors/deactivators of cellulase enzymes from lignocellulosic biomass Enzym Microb Technol 2011 48 408 415 10.1016/j.enzmictec.2011.01.007 22112958

[B7] Almeida JRM Modig T Petersson A Hahn-Hägerdal B Liden G Gorwa-Grauslund MF Increased tolerance and conversion of inhibitors in lignocellulosic hydrolysates by *Saccharomyces cerevisiae* J Chem Technol Biotechnol 2007 82 340 349 10.1002/jctb.1676

[B8] Koppram R Tomas-Pejo E Xiros C Olsson L Lignocellulosic ethanol production at high-gravity: challenges and perspectives Trends Biotechnol 2014 32 46 53 10.1016/j.tibtech.2013.10.003 24231155

[B9] Jorgensen H Vibe-Pedersen J Larsen J Felby C Liquefaction of lignocellulose at high-solids concentrations Biotechnol Bioeng 2007 96 862 870 10.1002/bit.21115 16865734

[B10] Mohagheghi A Tucker M Grohmann K Wyman C High solids simultaneous Saccharification and fermentation of pretreated wheat straw to ethanol Appl Biochem Biotechnol 1992 33 67 81 10.1007/BF02950778

[B11] Zhang MJ Wang F Su RX Qi W He ZM Ethanol production from high dry matter corncob using fed-batch simultaneous saccharification and fermentation after combined pretreatment Bioresour Technol 2010 101 4959 4964 10.1016/j.biortech.2009.11.010 20004092

[B12] Hoyer K Galbe M Zacchi G Effects of enzyme feeding strategy on ethanol yield in fed-batch simultaneous saccharification and fermentation of spruce at high dry matter Biotechnol Biofuels 2010 3 14 10.1186/1754-6834-3-14 20579340 PMC2908074

[B13] Olofsson K Bertilsson M Liden G A short review on SSF - an interesting process option for ethanol production from lignocellulosic feedstocks Biotechnol Biofuels 2008 1 7 10.1186/1754-6834-1-7 18471273 PMC2397418

[B14] Rudolf A Alkasrawi M Zacchi G Liden G A comparison between batch and fed-batch simultaneous saccharification and fermentation of steam pretreated spruce Enzym Microb Technol 2005 37 195 204 10.1016/j.enzmictec.2005.02.013

[B15] Taherzadeh MJ Niklasson C Lidén G Conversion of dilute-acid hydrolyzates of spruce and birch to ethanol by fed-batch fermentation Bioresour Technol 1999 69 59 66 10.1016/S0960-8524(98)00169-2

[B16] Öhgren K Bengtsson O Gorwa-Grauslund MF Galbe M Hahn-Hägerdal B Zacchi G Simultaneous saccharification and co-fermentation of glucose and xylose in steam-pretreated corn stover at high fiber content with *Saccharomyces cerevisiae* TMB3400 J Biotechnol 2006 126 488 498 10.1016/j.jbiotec.2006.05.001 16828190

[B17] Olofsson K Rudolf A Lidén G Designing simultaneous saccharification and fermentation for improved xylose conversion by a recombinant strain of *Saccharomyces cerevisiae* J Biotechnol 2008 134 112 120 10.1016/j.jbiotec.2008.01.004 18294716

[B18] Koppram R Nielsen F Albers E Lambert A Wannström S Welin L Zacchi G Olsson L Simultaneous saccharification and co-fermentation for bioethanol production using corncobs at lab, PDU and demo scales Biotechnol Biofuels 2013 6 2 10.1186/1754-6834-6-2 23311728 PMC3598390

[B19] Lin FM Qiao B Yuan YJ Comparative proteomic analysis of tolerance and adaptation of ethanologenic *Saccharomyces cerevisiae* to furfural, a lignocellulosic inhibitory compound Appl Environ Microbiol 2009 75 3765 3776 10.1128/AEM.02594-08 19363068 PMC2687285

[B20] Pinel D D’Aoust F del Cardayre SB Bajwa PK Lee H Martin VJJ *Saccharomyces cerevisiae* genome shuffling through recursive population mating leads to improved tolerance to spent sulfite liquor Appl Environ Microbiol 2011 77 4736 4743 10.1128/AEM.02769-10 21622800 PMC3147380

[B21] Palmqvist E Grage H Meinander NQ Hahn-Hägerdal B Main and interaction effects of acetic acid, furfural, and p-hydroxybenzoic acid on growth and ethanol productivity of yeasts Biotechnol Bioeng 1999 63 46 55 10.1002/(SICI)1097-0290(19990405)63:1<46::AID-BIT5>3.0.CO;2-J 10099580

[B22] Larsson S Quintana-Sainz A Reimann A Nilvebrant NO Jönsson LJ Influence of lignocellulose-derived aromatic compounds on oxygen-limited growth and ethanolic fermentation by *Saccharomyces cerevisiae* Appl Biochem Biotechnol 2000 84–6 617 632 10.1385/abab:84-86:1-9:617 10849822

[B23] Koppram R Albers E Olsson L Evolutionary engineering strategies to enhance tolerance of xylose utilizing recombinant yeast to inhibitors derived from spruce biomass Biotechnol Biofuels 2012 5 32 10.1186/1754-6834-5-32 22578262 PMC3408370

[B24] Larsson S Reimann A Nilvebrant NO Jönsson LJ Comparison of different methods for the detoxification of lignocellulose hydrolyzates of spruce Appl Biochem Biotechnol 1999 77–9 91 103

[B25] Vonsivers M Zacchi G Olsson L Hahn-Hägerdal B Cost-analysis of ethanol-production from willow using recombinant *Escherichia coli* Biotechnol Prog 1994 10 555 560 10.1021/bp00029a017 7765380

[B26] Alkasrawi M Rudolf A Liden G Zacchi G Influence of strain and cultivation procedure on the performance of simultaneous saccharification and fermentation of steam pretreated spruce Enzym Microb Technol 2006 38 279 286 10.1016/j.enzmictec.2005.08.024

[B27] Hoyer K Galbe M Zacchi G The effect of prehydrolysis and improved mixing on high-solids batch simultaneous saccharification and fermentation of spruce to ethanol Process Biochem 2013 48 289 293 10.1016/j.procbio.2012.12.020

[B28] Hoyer K Galbe M Zacchi G Production of fuel ethanol from softwood by simultaneous saccharification and fermentation at high dry matter content J Chem Technol Biotechnol 2009 84 570 577 10.1002/jctb.2082

[B29] Liu K Lin XH Yue J Li XZ Fang X Zhu MT Lin JQ Qu YB Xiao L High concentration ethanol production from corncob residues by fed-batch strategy Bioresour Technol 2010 101 4952 4958 10.1016/j.biortech.2009.11.013 20004568

[B30] Daran-Lapujade P Daran JM van Maris AJA de Winde JH Pronk JT Chemostat-based micro-array analysis in Baker’s yeast Adv Microb Physiol 2009 54 257 311 18929070 10.1016/S0065-2911(08)00004-0

[B31] Kristensen JB Felby C Jorgensen H Yield-determining factors in high-solids enzymatic hydrolysis of lignocellulose Biotechnol Biofuels 2009 2 11 10.1186/1754-6834-2-11 19505292 PMC2699335

[B32] Qing Q Yang B Wyman CE Xylooligomers are strong inhibitors of cellulose hydrolysis by enzymes Bioresour Technol 2010 101 9624 9630 10.1016/j.biortech.2010.06.137 20708404

[B33] Tejirian A Xu F Inhibition of enzymatic cellulolysis by phenolic compounds Enzym Microb Technol 2011 48 239 247 10.1016/j.enzmictec.2010.11.004 22112906

[B34] Verduyn C Postma E Scheffers WA Vandijken JP Effect of benzoic-acid on metabolic fluxes in yeasts - a continuous-culture study on the regulation of respiration and alcoholic fermentation Yeast 1992 8 501 517 10.1002/yea.320080703 1523884

